# Relationship between the expressions of DLL3, ASC1, TTF-1 and Ki-67: First steps of precision medicine at SCLC

**DOI:** 10.18632/oncotarget.28660

**Published:** 2024-10-11

**Authors:** Samuel Silva, Juliana C. Sousa, Cleto Nogueira, Raquel Feijo, Francisco Martins Neto, Laura Cardoso Marinho, Guilherme Sousa, Valeria Denninghoff, Fabio Tavora

**Affiliations:** ^1^Department of Pathology, Faculty of Medicine, Federal University of Ceará, Fortaleza (Ceará), Brazil; ^2^ARGOS Laboratory, Fortaleza (Ceará), Brazil; ^3^Messejana Heart and Lung Hospital, Fortaleza (Ceará), Brazil; ^4^Molecular Oncology Clinical Lab, University of Buenos Aires (UBA)—National Council for Scientific and Technical Research (CONICET), Buenos Aires, Argentina; ^5^Liquid Biopsy and Cancer Interception Unit, GENYO, Centre for Genomics and Oncological Research (Pfizer/University of Granada/Andalusian Regional Government), Granada, Spain

**Keywords:** DLL3, pathology, biomarkers, qupath, small cell carcinoma

## Abstract

This study presents an observational, cross-sectional analysis of 64 patients diagnosed with small cell lung cancer (SCLC) at a reference laboratory for thoracic pathology between 2022 and 2024. The primary objective was to evaluate the expression of Delta-like ligand 3 (DLL3) and other neuroendocrine markers such as Chromogranin, and Synaptophysin, utilizing both traditional immunohistochemistry and digital pathology tools. Patients were primarily older adults, with a median age of over 71, and most biopsies were obtained from lung parenchyma. Immunohistochemistry (IHC) was performed using specific monoclonal antibodies, with DLL3 showing variable expression across the samples. Notably, DLL3 was expressed in 72.3% of the cases, with varied intensities and a semi-quantitative H-score applied for more nuanced analysis. ASCL1 was expressed in 97% of cases, with the majority considered low-expressors. Only 11% had high expression. TTF-1, traditionally not a conventional marker for the diagnosis of SCLC, was positive in half of the cases, suggesting its potential as a biomarker. The study underscores the significant variability in the expression of neuroendocrine markers in SCLC, with implications for both diagnosis and potential therapeutic targeting. DLL3, particularly, shows promise as a therapeutic target due to its high expression rate in the cohort. The use of digital pathology software QuPath enhanced the accuracy and depth of analysis, allowing for detailed morphometric analysis and potentially informing more personalized treatment approaches. The findings emphasize the need for further research into the role of these markers in the management and treatment of SCLC, considering the poor prognosis and high mortality rate observed in the cohort.

## INTRODUCTION

Small cell lung cancer (SCLC) is an aggressive type of lung cancer that contributes to approximately 15% of lung cancer cases annually [1]. Patients with SCLC have a poor prognosis, with a 5-year survival rate ranging from 3 to 27%, depending on the stage of the disease [2]. SCLC is a highly proliferative lung cancer that is not amenable to surgery in most cases due to rapid growth, early spread, and a tendency to develop drug resistance and relapse [3]. Genes and genomics/proteomic modifications related to the development, plasticity, and progression of SCLC, which could be identified as possible biomarkers for targeted therapy of this deadly disease, were already described: TP53/RB1 (98%/91%), TP73 (13%), PI3K3CA (15%), PTEN (9%), FGFR1 (8%), Hedgehog Signaling Pathway (80%), MYC (20%), KMT2D (13%), and NOTCH1 signaling (25%) [4].

By July 19, 2022, 107 patients received Tarlatamab in dose exploration (0.003 to 100 mg; *n* = 73) and expansion (100 mg; *n* = 34) cohorts. The median progression-free and overall survival were 3.7 months (95% CI, 2.1 to 5.4) and 13.2 months (95% CI, 10.5 to not reached), respectively. Exploratory analysis suggests that selecting for increased DLL3 expression can increase clinical benefit [5]. On May 16, 2024, the US Food and Drug Administration (FDA) granted accelerated approval to tarlatamab-dlle for extensive-stage small cell lung cancer (ES-SCLC) with disease progression on or after platinum-based chemotherapy [6]. A phase 2 study was conducted on subjects with relapsed/refractory SCLC after two or more prior lines of treatment [7]. Efficacy, safety, tolerability, and pharmacokinetics of Tarlatamab were evaluated in 99 patients enrolled in DeLLphi-301, an open-label, multicenter, multi-cohort study [7]. Tarlatamab, administered at a 10-mg dose every two weeks, showed antitumor activity with durable objective responses and promising survival outcomes in patients with previously treated SCLC. No new safety signals were identified [7]. Tarlatamab (AMG 757) is the first DLL3-targeting bispecific T-cell engager therapy that activates a patient’s T cells to attack DLL3-expressing tumor cells, which is a bispecific T-cell engager molecule that binds both DLL3 and CD3, leading to T-cell-mediated tumor lysis [8].

DLL3 is a protein that plays a critical role in the Notch signaling pathway, which is involved in cell differentiation, proliferation, and apoptosis [9, 10]. In humans, DLL3 is predominantly expressed in neuroendocrine tissues. It has been aberrantly expressed on the surface of up to 80–85% of SCLC cells and minimally expressed in normal tissues, making it a compelling therapeutic target [5], such as in other neuroendocrine carcinomas [10, 11]. It is expressed both in the cytoplasm and in the membrane of SCLC cells [12]. Despite the growing body of knowledge on the role of DLL3 in lung cancer, there remains a significant gap in our understanding of the actual expression rate of DLL3 when assessed by immunohistochemistry (IHC) in routine clinical laboratories. In a real-world study of DLL3 as an SCLC therapeutic target, positive DLL3 expression (defined as ≥25% of tumor cells) was identified in 895/1050 (85%) patients with one specimen and evaluable DLL3 expression; 719/1050 (68%) patients had high DLL3 expression (defined as ≥75% of tumor cells). There was no significant difference in median overall survival from SCLC diagnosis for evaluable patients with non-missing data based on DLL3 expression (negative DLL3 expression (*n* = 139), 9.5 months; positive DLL3 expression (*n* = 747), 9.5 months; all evaluable patients (*n* = 893, 9.5 months) [13]. With the advent of anti-DLL3 therapies, studies of interrelationships between different molecules still need to be included, such as thyroid transcription factor-1 (TTF-1), which is involved in the differentiation of lung epithelial cells and is commonly expressed in high-grade lung and neuroendocrine adenocarcinomas, or Ki-67 protein (MKI67) which is a cellular marker for proliferation, found in the nucleus of cancer cells that are actively growing and dividing [14, 15]. These relationships could provide insights into the tumor biology of SCLC and rare tumors such as the Large-Cell Neuroendocrine Carcinomas (LCNEC), representing 1–3% of all primary lung cancers, and potentially guide treatment decisions and prognostication in a clinical setting [16, 17].

In this study, the qualitative and quantitative protein expression of DLL3, ASCL1, TTF-1, and Ki-67 was retrospectively analyzed by digital pathology in patients with SCLC, and this expression was linked to median overall survival using a multivariate mathematical model.

## RESULTS

### Patients’ characteristics

Sixty-four cases were included (mean age 71 ± 10), with a balanced relation between gender (32 females and 32 males, Table 1). The mean age for males was 72 ± 10 years, and for females, 70 ± 10 years (*p* = 0.460). Most patients were older than 60 (54 patients, 84,4%), as depicted in the population pyramid (Table 1).

**Table 1:** Detailed patient demographics and quantitative analysis of neuroendocrine biomarker expression (KI-67, Chromogranin A, Synaptophysin, CD56, TTF1, ASCL1, and DLL3) in small cell lung cancer (SCLC) cohort

Characteristics	Results		
*Gender, n (%)*			
Female	32 (50%)		
Male	32 (50%)		
*Age at diagnosis, Average (range)*	71 (41–43)		
*Half-life overall survival (days)*	136.19		
*KI-67*			
Global, *median* (range)	80.0 (40–97.20)		
*Chromogranin A*			
Class N (%)	Negative	Positive	
	11 (29.7%)	26 (70.3%)	
*Synatophisin*			
Class N (%)	Negative	Positive	
	6 (16.2%)	31 (83.8%)	
CD56			
Class N (%)	Negative	Positive	
	2 (5.6%)	34 (94.4%)	
*TTF1*			
H-score, median (range)	37.30 (0–296.52)		
Quantification, median (range)	17.25 (0–99.90)		
Classification 1 Quantification, *N* (%)	Negative (TTF1 = 0)	Positive (TTF1≥1)	
	31 (48%)	33 (52%)	
Classification 2 Quantification, *N* (%)	Negative (TTF1 = 0)	Low (1 ≤ TTF1 ≤ 49)	High (50 ≤ TTF1 ≤ 100)
	31 (48%)	4 (6%)	29 (45%)
Classification 3 H-score, *N* (%)	Negative (TTF1 = 0)	Low (1 ≤ TTF1 ≤ 149)	High (150 ≤ TTF1 ≤ 300)
	31 (48%)	12 (19%)	21 (33%)
*ASCL1*			
H-score, median (range)	57.08 (0.01–268.66)		
Quantification, median (range)	51.10 (0–99,90)		
Classification 1 Quantification	Negative (ASCL1 = 0)	Positive (ASCL1≥1)	
	2 (3%)	62 (97%)	
Classification 2 Quantification	Negative (ASCL1 = 0)	Low (1 ≤ ASCL1 ≤ 49)	High (50 ≤ ASCL1 ≤ 100)
	2 (3%)	28 (44%)	34 (53%)
Classification 3 H-score	Negative (ASCL1 = 0)	Low (1 ≤ ASCL1 ≤ 149)	High (150 ≤ ASCL1 ≤ 300)
	2 (3%)	55 (86%)	7 (11%)
*DLL3*			
H-score, median (range)	57.08 (0–289)		
Quantification, median (range)	51.10 (0–99.30)		
Classification 1 Quantification	Negative (DLL3 = 0)	Positive (DLL3≥1)	
	18 (28%)	46 (72%)	
Classification 2 Quantification	Negative (DLL3 = 0)	Low (1 ≤ DLL3 ≤ 49)	High (50 ≤ DLL3 ≤ 100)
	18 (28%)	27 (42%)	19 (30%)
Classification 3 H-score	Negative (DLL3 = 0)	Low (1 ≤ DLL3 ≤ 149)	High (150 ≤ DLL3 ≤ 300)
	18 (28%)	42 (66%)	5 (8%)

The majority of cases were biopsied from lung parenchyma, either by transbronchial/endobronchial biopsies or transthoracic CT-guided procurement (56 cases, 90,3%). Four cases were pleural biopsies, and two were metastasis in lymph nodes.

Chromogranin was positive in 70,3% of cases, with 15,4% showing 1+ intensity, 19,2% 2+ intensity, and 23,1% 3+ intensity. Synaptophysin was positive in 83,8% of cases, with 24,0% showing 1+ intensity, 20,0% 2+ intensity, and 32,0% 3+ intensity. CD56 was positive in 94,4% of cases, and its intensity was not evaluated (Table 1). All cases had at least one classical neuroendocrine marker positive and conventional small-cell carcinoma morphology.

Fifteen patients (18%) were followed by palliative care and did not receive chemotherapy. All remaining patients included in the study received standard chemotherapy for small-cell neuroendocrine carcinoma. The follow-up was complete until the patients died from the disease. The mean overall survival was 77.5 days with a 95% confidence interval of 36 to 116 days (Table 1), with a maximum of 557 days.

### TTF-1 expression

While TTF-1 is not usually considered a conventional marker for diagnosing small cell carcinoma in most centers, it is positive in most of them. In the current cohort, it was positive in 33 cases (52%) and negative in 31 cases (48%) (Table 1). The percentage of tumor cells with TTF-1 averaged 39.6% (SD 43.4). Eleven (11, 18.3%) had 100% of TTF-1 positivity. When assigned a histologic score of percentage versus intensity of positivity, cases had an H-score median of 37,30 (SD 110,08). Twenty-one cases (21, 33%) had an H-score of 150 or higher (Table 1).

### Ki67 expression

**Figure 1:**
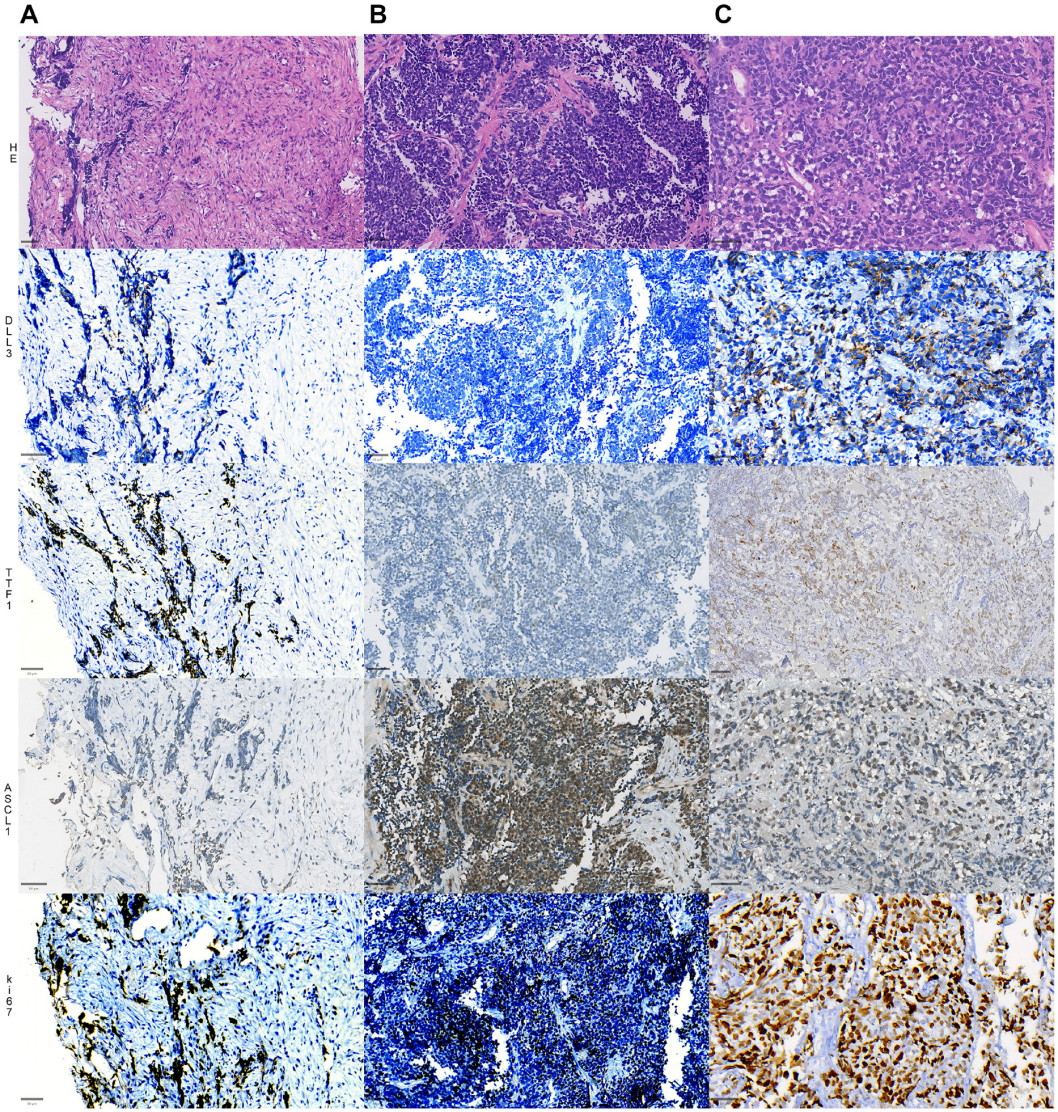
Representative slides of 3 different cases of SCLC (**A**–**C**), showing distinct morphology (top panel, HE sections) and expressions of DLL3 in the second row, TTF1 in the third row, ASCL1 expression in the fourth row and ki67 index in the fifth and final row. The first cases (1st column) shows a moderate expression of DLL3, strong positivity for TTF1, weak and focal positivity for ASCL1; the second case (second column) shows negative DLL3, negative TTF1, strong and diffuse nuclear expression for ASCL1, while the third case (3rd column) shows strong positivity for DLL3, TTF1 and ASCL1. All cases show a high ki-67 proliferative index. Bars = 50 µM (micrometers).

Ki67 was positively expressed in all cases diagnosed with small cell carcinoma due to its high proliferation rate (Figure 1). In the cohort, Ki67 showed positive expression in 100% of the cases, with an average percentage of positive cells of 73.73% (SD: 15.80). The case with the highest expression exhibited an immunohistochemical positivity of 97.20%, while the case with the lowest expression showed positivity in 40% of the cells (Table 1).

### ASCL1 expression

Tissue was available for the study of ASCL1 in 64 cases (Figure 1). The H-score had a median of 57,08 (SD 54.55). Only two cases (3%) were completely negative for this antibody, while the majority (55 cases, 86%) had an H-score of 10–150 and were considered low-expressors. Seven cases (7, 11%) were considered high expression. Only one case (1.4%) had an H-score of more than 250 (Table 1).

### DLL3 expression

**Figure 2:**
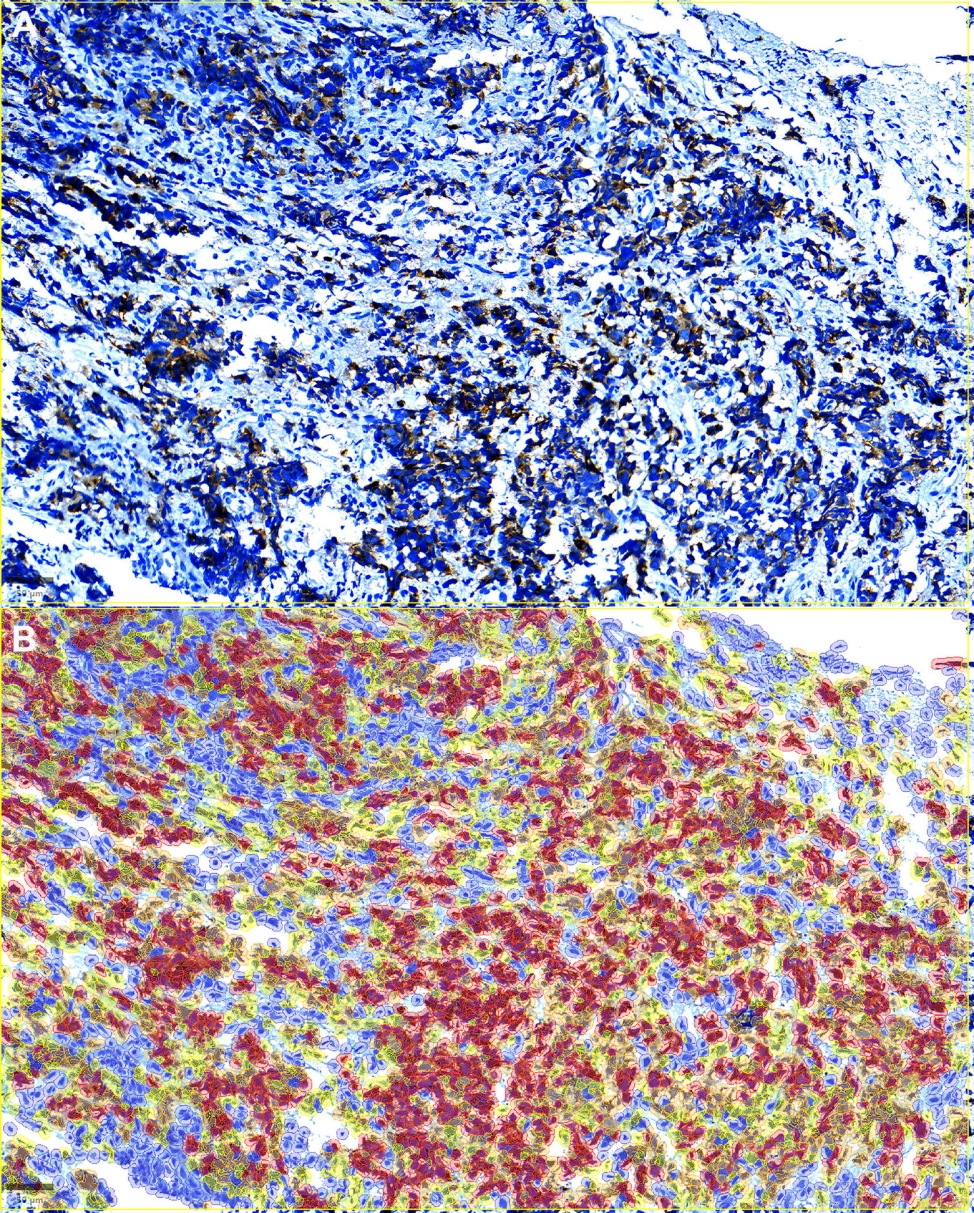
Qupath H-score calculation and analyses. Top (**A**). Representative image of a case showing DLL3 positivity in tumor cells. Note the strong cytoplasmic and membranous staining. Bottom (**B**) Representative image form the same field from above show cell staining intensity for the indicated DLL3 protein (DAB staining calculation). Cell staining intensity is represented as follows: negative (blue), low (yellow), medium (orange), and high (red). The H-score is calculated based on the percentage of positive cells by the staining of low, medium and high intensity. Bar = 50 µM (micrometers).

DLL3-positive SCLC tissue was used as a positive control, and DLL3-negative lung adenocarcinoma tissue was used as a negative control. As per previously published data (Figure 2), the staining pattern was cytoplasmic and membranous (Figure 1) [13, 18].

Forty-six (46, 72%) had some expression of DLL3 (18 negative, 28%). Nineteen cases (30%) expressed DLL3 in less than 50% of tumor cells, while 27 (42%) expressed it in more than 50% of cells. When the h-score was calculated, only five cases (8%) scored above 150 (Table 1).

### Association between DLL3, ASC1, TTF-1 and Ki-67 immunoexpression

**Figure 3:**
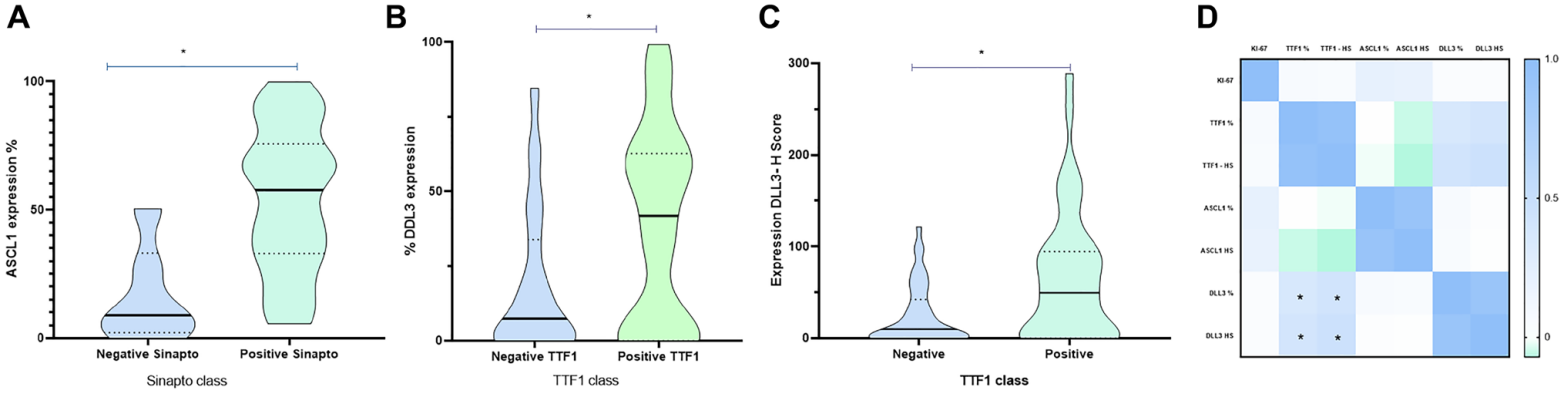
Immunoexpression of TTF1, ASCL1 and DLL3 in patients with SLCL. (**A**) Association of ASCL1 immunoexpression according to synaptophysin positivity. Patients with a positive synaptophysin stain are more likely to have greater ASCL1 expression, than patients with a negative synaptophysin. (**B**) DLL3 expression (percentage of positive cells) according to TTF1 expression in patients with SLCL. (**C**) DLL3 expression (H-score) according to TTF expression in patients with SLCL. (**D**) Correlation Biomarker, TTF1 and DLL3 correlate in patients with SCLC. ^*^ *p* < 0,05.

Both TTF-1 and DLL3 were evaluated by the percentage of positive cells and H-score. ASCL1 was evaluated by H-score. As expected, ASCL1 expression was strongly associated with synaptophysin positivity (*p* = 0,003) (Figure 3A, Table 2) ASCL1 expression did not have any differences regarding age, Ki-67 positivity, chromogranin or TTF-1 expression (Table 2). DLL3 expression was strongly associated with TTF-1 positivity (Figure 3B, 3C and Table 2). Tumors that were positive for TTF-1 had a higher percentage of DLL-3 expression both in percentage as well as in H-score (*p* < 0.001). The correlation between biomarkers TTF-1 and DLL3 was positive demonstrated in Figure 3D.

**Table 2:** Association biomarkes KI67, TTF1, DLL3 and ASCL1 and characteristics in patients SCLC

		KI67		TTF1 Quant		TTF1 HS		ASCL1 Quant		ASCL1 HS		DLL3 Quant		DLL3 HS	
		Mediana	*P*-value	Mediana	*P*-value	Mediana	*P*-value	Mediana	*P*-value	Mediana	*P*-value	Mediana	*P*-value	Mediana	*P*-value
**Age class1**	≤60	80,00	0,341	11,400	0,579	29,250	0,551	73,950	0,536	83,955	0,582	33,440	0,725	56,655	0,536
	≥61	79,70		29,300		40,900		50,700		56,345		15,750		25,865	
**Age class 2**	≤74	80,00	0,299	54,700	0,129	71,200	0,481	62,270	0,302	64,680	0,119	13,340	0,200	14,940	0,425
	≥75	70,00		0,000		0,000		49,110		51,280		29,400		40,240	
**Sex**	Female	79,70	0,393	53,000	0,314	70,100	0,512	51,780	0,201	56,825	0,148	24,000	0,444	32,850	0,684
	Male	80,00		0,000		0,000		51,100		57,080		15,750		25,865	
**Chromo A**	Negative	80,00	0,229	54,700	0,707	65,700	0,285	44,950	0,481	51,280	0,402	2,810	0,707	3,270	0,366
	Positive	80,00		51,100		75,400		49,010		55,380		14,950		21,675	
**SyP**	Negative	80,00	0,888	50,600	0,952	69,350	0,888	8,854	**0,004**	9,075	**0,005**	5,145	0,533	5,745	0,615
	Positive	80,00		54,700		69,000		53,110		55,700		13,500		13,700	
**TTF1 Quant class 1**	Negative	80,00	0,770	0,000	*	0,000	*	67,290	0,097	74,540	0,103	13,340	0,068	14,940	0,071
	Positive	80,00		90,400		182,100		44,950		55,060		35,000		46,800	
**TTF1 Quant class 2**	Negative (0)	80,00	0,563	0,000	*	0,000	*	67,290	0,055	74,540	0,062	13,340	0,189	14,940	0,199
	Low ( 1–49)	70,00		34,850		63,750		10,965		11,400		21,500		35,550	
	High (50–100)	80,00		92,100		237,600		47,570		55,430		40,600		52,110	
**TTF1 HS class 3**	Negative	80,00	0,634	0,000	*	0,000	*	67,290	0,181	74,540	0,226	13,340	**0,008**	14,940	**0,009**
	Low (1–149)	75,00		55,000		81,000		37,490		41,030		5,145		5,745	
	High (149–300)	80,00		96,100		249,520		44,950		55,430		55,690		79,300	
**ASCL1 Quant class 1**	Negative	66,90	0,571	49,800	0,620	137,300	0,693	0,045	*	0,050	*	0,000	0,071	0,000	0,063
	Positive	80,00		17,250		37,300		51,525		57,695		18,600		30,025	
**ASCL1 Quant class 2**	Negative (0)	66,90	0,369	49,800	0,537	137,300	0,588	0,045	*	0,050	*	0,000	0,152	0,000	0,158
	Low (1–49)	70,00		51,100		70,100		27,740		31,050		24,250		35,690	
	High (50–100)	80,00		0,000		0,000		74,410		86,980		14,950		28,540	
**ASCL1 HS class 3**	Negative (0–10)	80,00	0,309	0,000	0,578	0,000	0,645	7,050	*	7,670	*	10,200	0,412	11,200	0,322
	Low (11–150)	74,70		49,500		70,100		52,455		58,925		23,500		37,770	
	High (150–300)	80,00		0,000		0,000		95,840		172,960		24,390		27,430	
**DLL3 Quant - class 1**	Negative	75,00	0,640	27,350	0,912	32,850	0,601	50,030	0,464	54,135	0,446	0,000	*	0,000	*
	Positive (1–100)	80,00		17,250		37,300		51,525		57,695		37,800		52,655	
**DLL3 Quant - class 2**	Negative	75,00	0,837	27,350	**0,011**	32,850	**0,003**	50,030	0,762	54,135	0,748	0,000	*	0,000	*
	Low (1–49)	80,00		0,000		0,000		53,110		57,170		16,400		24,300	
	High (50–100)	80,00		83,800		182,100		50,450		58,220		64,930		95,200	
**DLL3 HS - class 2**	Negative	80,00	0,848	54,700	**0,019**	65,700	**0,006**	49,110	0,995	51,280	0,992	0,000	*	0,000	*
	Low (1–149)	79,70		0,000		0,000		52,455		58,925		33,440		43,325	
	High (150–300)	80,00		99,300		242,900		37,150		39,150		94,310		170,930	

Abbreviations: CgA: Chromogranin A; Syp: Synapthophysin; TTF1: Thyreoid transcription factor 1; DLL3: Delta-like ligand 3; ASCL1: Achaete-scute homolog; HS: HScore.

### Survival and multivariate analyses

**Figure 4:**
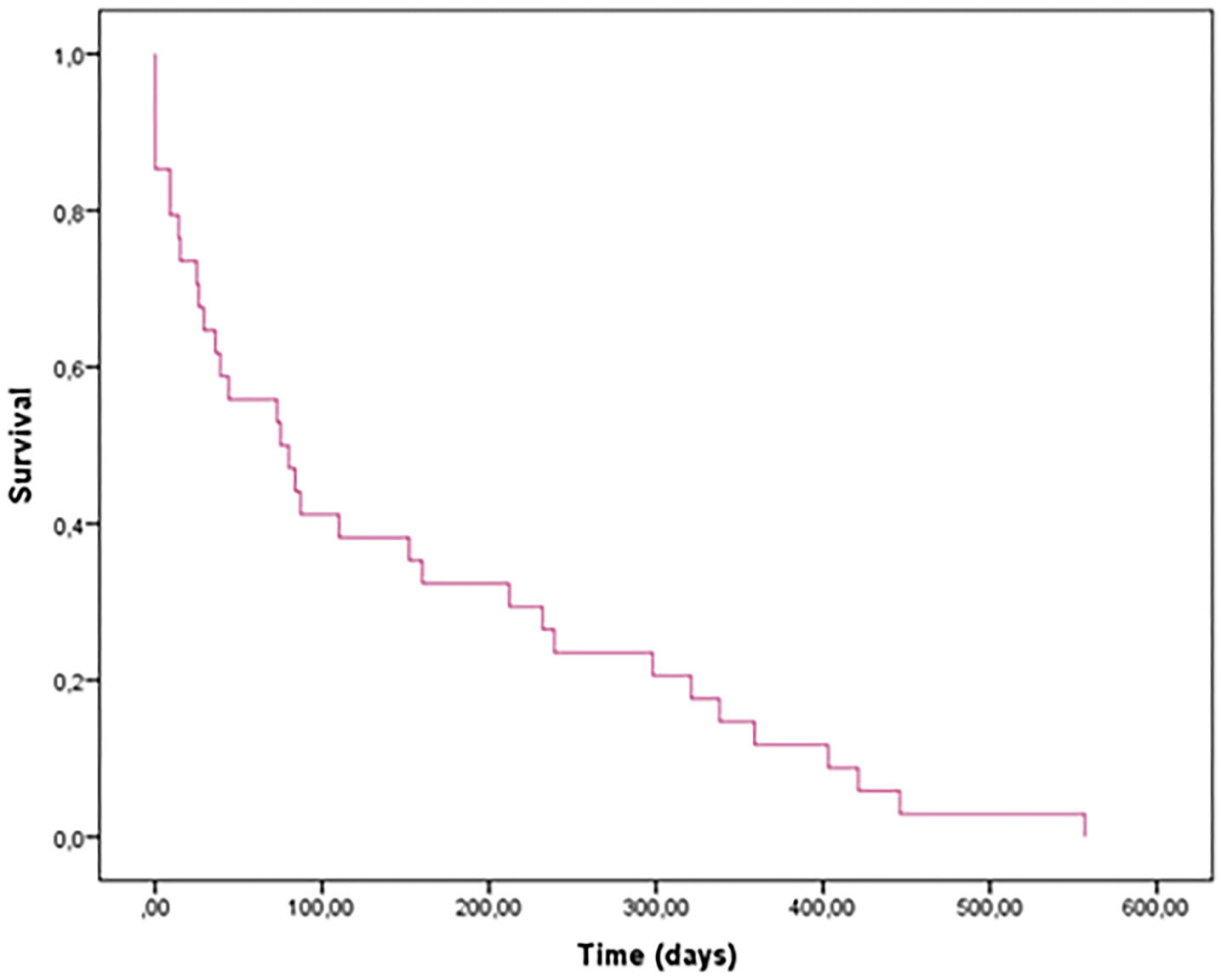
Global survival of all patients with DLL3.

**Figure 5:**
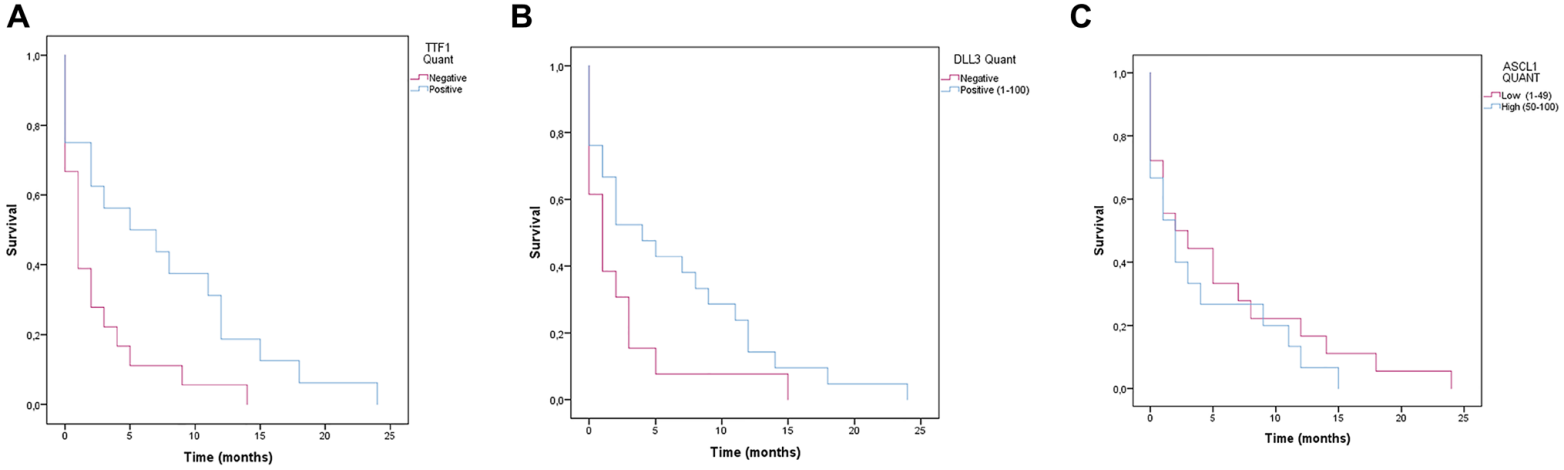
Survival analysis (Kaplan-Meier) of patients with SCLC. (**A**) TTF1 negative patients have a lower survival rate than TTF1 positive SCLC patients (Log rank *p* = 0.014) and may be associated with an unfavorable prognosis with worse outcome in patients with SCLC. (**B**) DLL3 negative patients have a lower survival rate than DLL3 positive SCLC patients) (*p* = 0.073). (**C**) Survival analysis of patients with SCLC according to ASCL1 immunoexpression (*p* = 0,458).

The mean global survival of all patients included in the study was 77.5 days (Figure 4). Age, sex, and all conventional neuroendocrine markers did not correlate with overall survival. Using Cox regression, epidemiological variables, as well as TTF-1 and DLL3 expression were tested. It was observed that TTF1 negative patients are a marker of worse prognosis in patients with SCLC compared to patients with positive expression (*p* = 0.014) (Figure 5A). [...] DLL3 and ASCL1 did not have any correlation with overall survival (Figure 5B, 5C).

## DISCUSSION

Precision medicine is an innovative approach to disease prevention and treatment that considers differences in people’s genes, injuries, environments, and lifestyles to target the right therapies to the right patients at the right time. In oncology, precision medicine uses genetic and molecular information, tailoring treatment on a single patient profile, optimizing efficacy, and minimizing toxicities [19]. This approach is revolutionizing lung cancer diagnosis and treatment. However, despite being widely adopted, its benefit in clinical practice still remains to be fully elucidated [20].

SCLC continues to carry a poor prognosis, with a five-year survival rate of 3.5% and a 10-year survival rate of 1.8% [21]. The pathogenesis remains unclear, and no known predictive or diagnostic biomarkers exist. Delta-like ligand 3 (DLL3) is an inhibitory Notch ligand that is highly expressed in small cell lung cancer (SCLC) and has been identified as a potential therapeutic target [14, 22, 23]. DLL3 expression is not commonly found in normal adult tissues, which makes it an attractive target for anti-cancer therapies [22]. High DLL3 expression has been associated with poor prognosis in SCLC patients, suggesting its potential role as a prognostic biomarker [24, 25]. However, the prognostic significance of DLL3 expression in SCLC remains controversial, with some conflicting studies indicating a potential association between high DLL3 expression and overall survival [14].

Therapeutic strategies targeting DLL3, such as antibody-drug conjugates (ADCs), bispecific T-cell engagers, and chimeric antigen receptor (CAR) T-cell therapies, are under development [5, 26, 27]. Rovalpituzumab tesirine (Rova-T), an ADC targeting DLL3, has been evaluated in clinical trials, although it did not meet the expected outcomes in Phase III trials [23]. Other investigational therapies, including bispecific T-cell engagers like tarlatamab (AMG 757) and CAR T-cell therapies targeting DLL3, have shown promise in preclinical models and early clinical trials [28, 29].

The study conducted by Furuta et al. provides critical insights into the expression of these proteins in surgically resected SCLC samples [15]. The study reveals a high prevalence of DLL3 and ASCL1 expression in SCLC patients, with ASCL1 expression detected in 83% of the evaluated samples. These findings agree with our paper, which showed 90% positivity of ASCL1. This high expression rate aligns with DLL3’s potential role in the disease’s pathology and supports the development of DLL3-targeted therapies. The positive correlation between DLL3 and ASCL1 expressions further underscores their interconnected roles in SCLC’s molecular landscape, suggesting that interventions targeting these pathways could offer new avenues for treatment [15]. Their study also explores the prognostic implications of DLL3 and ASCL1 expression, finding no direct association with patient survival. Similarly to their findings, in out cohort we have not found any direct association of ASCL1 and DLL3 with the overall survival, although we found a relation between positive TTF1 and a better survival rate (quantified by percentage of positive cells). These findings may be important in establishing practical protocols for scoring these immunohistochemical studies and selecting patients that may benefit from targeted therapies.

Similarly, another recent study demonstrated that high DLL3 and ASCL1 expression was associated with certain morphological features in LCNECs and SCLCs, and in early-stage patients without metastasis who underwent chemotherapy, high expression of both DLL3 and ASCL1 was linked to a better prognosis and a lower risk of death [30]. Furthermore, DLL3 expression in LCNEC was associated with the expression of ASCL1 and neuroendocrine markers, suggesting a relationship between DLL3 expression and the neuroendocrine profile of these tumors [18]. These findings suggest that DLL3 and ASCL1 are not only correlated in their expression but may also be involved in the neuroendocrine phenotype of lung neuroendocrine tumors and could serve as potential therapeutic targets or prognostic indicators in these diseases. Specifically, ASCL1-positive/DLL3-high tumors may represent a subgroup of SCLC with unique vulnerabilities to DLL3-targeted therapies. Further research is warranted to validate these findings and explore the clinical utility of ASCL1/DLL3 co-expression as a predictive biomarker for therapeutic response.

In adenocarcinomas, TTF-1 has been shown to play a significant role in the pathogenesis of lung cancer, being expressed in 69–80% of lung adenocarcinoma cases. Clinically, TTF-1 expression is a diagnostic tool for identifying the histological type of lung cancer, distinguishing primary lung adenocarcinomas from metastatic forms, and acting as a prognostic indicator. Studies have shown that patients with positive TTF-1 expression exhibit longer overall survival (OS) in stage I lung adenocarcinoma [31, 32].

Small Cell Lung Cancer (SCLC), typically characterized as an undifferentiated cancer, exhibits TTF-1 positivity in 80–90% of cases, indicating a function beyond epithelial cell differentiation. Evidence of TTF-1 expression in non-pulmonary small cell cancers, such as aggressive small cell prostate cancer, supports its association with neuroendocrine differentiation and aggressive tumor behavior rather than characteristics of terminal respiratory unit cells [33–35]. In our samples, of interest, was the association of TTF-1 score with DLL3 expression, showing a potential role in TTF-1 as a differentiation and mechanistic marker, much more than only a diagnostic one.

The significant prevalence of DLL3 and ASCL1 expression in early-stage SCLC, as highlighted by Furuta et al. and corroborated by our findings, underscores their potential as therapeutic targets and prognostic biomarkers [15]. Our study further expands upon this, revealing a correlation between TTF1 positive expression and improved survival outcomes, emphasizing the importance of standardized scoring protocols for these immunohistochemical markers. This may enable the identification of patient subgroups that could particularly benefit from DLL3-targeted therapies, potentially personalizing treatment approaches for SCLC.

Additionally, the intriguing association between TTF-1 expression and DLL3, as observed in our study, suggests a multifaceted role for TTF-1 beyond its established diagnostic utility. This finding may have implications for understanding the molecular underpinnings of SCLC and could inform the development of novel therapeutic strategies. Further investigations into the mechanistic link between TTF-1 and DLL3 could uncover new avenues for intervention in this aggressive disease.

Despite the promising insights and potential therapeutic implications highlighted in our study, there are several limitations that should be acknowledged. First, our study’s retrospective design may introduce selection bias, as it relies on previously collected data and samples, which may not be representative of the broader SCLC patient population. Additionally, the relatively small sample size limits the generalizability of our findings and may impact the statistical power to detect significant associations or differences in survival outcomes.

Furthermore, our study primarily focuses on the expression of DLL3 and ASCL1 in small SCLC samples, which may not fully capture the heterogeneity of SCLC, especially in that most cases are inoperable or treated with different modalities. The lack of longitudinal data to track changes in marker expression over time and in response to treatment is another limitation. Finally, the interpretation of immunohistochemical scoring can be subjective, and inter-observer variability might affect the consistency of the results, even with the attempted scoring protocols tried here. Future studies should aim to include larger, more diverse cohorts and incorporate prospective designs to validate these findings and enhance their clinical applicability.

In summary, our findings and corroborative studies present a compelling case for the significance of TTF1 in the clinical landscape of small-cell lung cancer. The evidence of a better survival rate in patients with high expression of these proteins, despite the generally poor prognosis associated with SCLC, indicates their potential utility as biomarkers and as focal points for targeted therapy. Future research should continue to explore the mechanistic pathways influenced by these proteins, emphasizing developing therapeutic strategies that can effectively exploit these targets. By advancing our understanding of DLL3 and ASCL1 within the broader context of lung cancer pathology, we can hope to refine diagnostic criteria and enhance the specificity and efficacy of treatment protocols, ultimately leading to improved survival rates and quality of life for patients afflicted by this formidable disease.

## MATERIALS AND METHODS

### Cohort description

This observational, cross-sectional, and analytical study had a cohort of sixty-four sequential patients recruited between May 2018 and November 2022. Biopsies were analyzed in a reference thoracic pathology laboratory. Data were collected from electronic medical records in the respective hospital units where each patient was diagnosed and followed up. Inclusion criteria were defined as adults over 18 years of age with transbronchial biopsy of a primary SCLC tumor confirmed by histological analysis, sufficient material for the study of HE, DLL3, ASCL1, TTF-1, and Ki-67, and clinical follow-up to death. Exclusion Criteria were under 18, insufficient material for IHC analysis, lack of clinical data, or loss of clinical follow-up. This protocol was reviewed and approved by the Research Ethics Committee at the Federal University of Ceará (Protocol CAAE 59399322.9.0000.5049). The study was conducted under the Good Clinical Practice Guidelines and the Helsinki Declaration.

### Immunohistochemistry

Each tumor formalin-fixed, and paraffin-embedded tissue block was sectioned at 2 µm. A hematoxylin and eosin (HE) staining was performed. Slides were stained with anti-DLL3-specific monoclonal antibody (dilution 1:100; clone EPR22592-18; cat. no. ab229902; Abcam, Cambridge, UK); anti-ASCL1 polyclonal antibody (dilution 1:200; cat. no. PA5-77868; Invitrogen, Massachusetts, USA); anti-TTF-1 specific monoclonal antibody (prediluted; clone 8G7G3/1; cat. no. 790-4398; Ventana Medical Systems, Inc.); and anti-Ki-67 monoclonal specific antibody (prediluted; clone 30-9; cat. no. 790-4286; Ventana Medical Systems, Inc.). We used the Ultraview DAB IHC Detection Kit (cat. no. 760–500; Ventana Medical Systems, Inc.), which includes a blocking reagent and a secondary antibody conjugated with polymer. Staining was performed using standard automated immunostaining equipment (Ultraview Benchmark Ventana; Ventana Medical Systems, Inc., Tucson, AZ, USA) according to the manufacturer’s protocol. Chromogranin, synaptophysin and Ki-67 had been previously performed for the diagnosis, and retrieved from the pathology files. IHC slides had a positive control tissue: glioblastoma for DLL3, neuroendocrine tumor for ASCL1, thyroid tissue for TTF-1, and tonsil tissue for Ki-67. Positive and negative control slides were included in each assay. The slides were analyzed by optical microscopy to evaluate the positive and negative controls.

### Digital pathology analysis

ASCL1, DLL3, TTF-1 and Ki-67 *s*lides were scanned using the KFBIO scanner equipment at 40x magnification. The SVS files were then imported to QuPath^®^ software v. 0.5.0 as “DAB Brightfield,” which allowed sample analysis. The files were loaded onto a project in QuPath software (QuPath source code, documentation, and links to the software download are available at https://qupath.github.io).

QuPath’s segmentation feature can detect thousands of cells, identify them as objects in a hierarchical manner below the annotation or cases, and measure cell morphology and biomarker expression simultaneously (12). QuPath has recently been used as annotation software in deep learning to distinguish small-cell from large-cell neuroendocrine lung cancer [36].

For each slide the stain vectors were recalibrated on “Estimate Stain Vector” with automatic calibration. The positive cell detection was performed by the nucleus evaluation according to default parameters; the nucleus staining intensity threshold was set as 0.1, and the cell expansion was set to default to 5 micrometers, which is the default measurement for cell cytoplasm expansion from the nucleus until it meets the neighboring cell. The DAB intensity threshold was standardized according to each marker. For DLL3, the “thresholdCompartmen” was set to be “Cytoplasm: DAB OD Mean,” and for ASCL1, Ki-67, and TTF-1 the “thresholdCompartmen” was set to be “Nucleus: DAB OD mean.”

For H-Score analysis, the intensity threshold parameters were set with three threshold points: the “thresholdPositive1” was set to 0.2, the “thresholdPositive2” was set to 0.4, and the “thresholdPositive3” was set to 0.6. The analysis was performed for each marker and the results were obtained as positive and negative, percentage and HScore. Figure 1 depicts an example of DLL3 expression in a tumor showing the deployment of QuPath algorithm to assess cells with zero, low, moderate and high expressions, which is color coded and curated by an experienced pathologist. Snapshots of representative images were exported to ImageJ for storage and illustrations (Figures 1 and 2), exported in high quality using TIFF extensions with 300 dpi and at least 5 inches in the shortest axis.

### Scoring criteria biomarkers

For DLL3, ASCL1, and TTF-1, IHC scoring was performed in two ways. First, the staining was semi-quantitatively evaluated using an immunohistochemical H-score (HS) method by an experienced thoracic pathologist and also by using a algorithm developed and of free access by QuPath [37–43]. The H-score method was applied based on the extent and intensity of cytoplasmic staining (1, 2, or 3) multiplied by the percentage of cells positive (proportion score), with a potential score ranging from 0 to 300.

The H-score is a classic semi-quantitative method used in pathology to assess the intensity and distribution of immunohistochemical staining in tissue samples. It is particularly valuable in research for evaluating the expression levels of various proteins within specific cells or tissue regions, which can be crucial for diagnosing and determining the prognosis of diseases, especially cancer. It has been used in several organ systems and cancer types, including oral squamous cancer, kidney cancer, breast cancer and lung cancer [37–43].

Over the past decade, several studies [37–45] have developed automated algorithms for the quantitative assessment of IHC images. However, significant efforts are still needed to improve quantification accuracy and efficiency [44–47]. More recently, several articles have automated the use of H-scoring to increase accuracy and reproducibility, using the QuPath software, as in the current study [48–51].

The second way was the analysis of the percentage of positive cells (0–100%). The cut-off of negative and positive, low and high, was according to each protein expression profile and was used as described in previous studies [45]. DLL3 and TTF-1 were considered positive if at least 1% of tumor cells had cytoplasmic and/or membranous on DLL3 and nuclear staining on TTF-1. Both proteins were considered low expression if positive in less than 50% of tumor cells, while high expression was assumed if the protein was positive in more than 50% of tumor cells. ASCL1 was considered positive if at least 10% of tumor cells had nuclear staining. ASCL1 – H-score patients ≤10 were considered negative, H-scores of 11–149 were considered low expressed, and 150–300 were considered high expressed.

Chromogranin and synaptophysin were considered positive if at least 5% of tumor cells had cytoplasmic and/or membranous staining. In addition, a semi-quantitative scoring of 1, 2, and 3 intensity of staining was estimated by at least one pathologist. CD56 staining was considered only as positive when shown a membranous staining, or negative [52, 53].

The most recent 2021 WHO classification identifies the three markers indicative of neuroendocrine (NE) differentiation: chromogranin A, synaptophysin, and CD56. In addition, it mentions INSM1 as a potential new marker [54]. Determining positivity for these markers lacks defined thresholds, necessitating consideration of morphological features. Chromogranin and synaptophysin are genuine indicators of NE differentiation, as they bind to epitopes present in neurosecretory granules or synaptic vesicles. In SCLC, focal positivity for chromogranin A in some tumor cells is diagnosed [55, 56].

### Statistical analysis

Univariate descriptive statistics were performed on the recollected data. Normal variables were reported by their mean and standard deviation, and non-normal counterparts by median and interquartile range; count data were reported by absolute frequency and percentage. Overall survival analysis included univariate Kaplan-Meier curves using different biomarker strata according to DLL3, ASCL1, and TTF-1 presence, expression levels, and gender. Multivariate analysis included a correlation plot over the numerical variables and Cox regression analysis using a backstep variable selection strategy.
